# Assessment of hemodynamics, blood gases, and lung histopathology of healthy Pig model on two different mechanical ventilators

**DOI:** 10.1016/j.heliyon.2022.e10736

**Published:** 2022-09-22

**Authors:** Kamal Hussein, Ahmed F. Ahmed, Magda M.A. Omar, Rania A. Galhom, Mostafa Salah, Ola Elrouby, Yasser Nassar

**Affiliations:** a Animal Surgery, Anesthesia, and Radiology Department, Faculty of Veterinary Medicine, Assiut University, Assiut, Egypt; b Human Anatomy & Embryology Department, Faculty of Medicine, Suez Canal University, Ismailia, Egypt; c Center of Excellence in Molecular and Cellular Medicine (CEMCM), Faculty of Medicine, Suez Canal University, Ismailia, Egypt; d Human Anatomy & Embryology Department, Faculty of Medicine, Badr University in Cairo (BUC), Cairo, Egypt; e Clinical Research Department, TCD MENA, Egypt; f Critical Care Medicine Department, Faculty of Medicine, Cairo University, Cairo, Egypt

**Keywords:** Arterial blood gases, Lung histopathology, Lung mechanics, Ventilators, Pigs

## Abstract

In response to COVID-19 global crisis and arising from social responsibility, efforts have been exerted to promptly research, develop and manufacture ICU ventilators locally to meet the spike in demand. This study ***aimed at***: Evaluating the safety and performance of a newly developed mechanical ventilator; EZVent compared to a commercial ventilator regarding hemodynamics, arterial blood gases (ABG), lung inflammatory markers, and histopathology in a healthy pig model using three different ventilation modes. ***Methods:*** Eight adult male pigs were anesthetized and randomly assigned into two equal groups: Commercial vent and EZVent group, the animals of which were ventilated using a standard commercial ventilator and EZVent, respectively. On every animal, three ventilation modes were tested, each mode for 30 min: CMV-VC, CMV-PC, and CPAP-PS modes. Vital signs, ECG, Lung Mechanics (LM), and ABG were measured before ventilation and after 30 min of ventilation of each mode. After animals’ euthanasia, histological examinations of lung samples including morphometric assessment of alveolar edema, alveolar wall thickening, and the mean number of inflammatory cellular infiltrate/cm^2^ of lung tissue were analyzed. TNF-α and Il-6 expression and localization in lung tissue were assessed by western blot and immunohistochemistry. ***Results:*** The vital signs, LM, ABG, morphometric analysis, and histopathological score during the different ventilation modes showed non-significant differences between the study groups. TNF-α and IL-6 were minimally expressed in the bronchiolar epithelium and the alveolar septa. Their increased expression level was insignificant. ***Conclusion:*** EZVent is equivalent to the commercial ventilator regarding its safety and efficacy.

## Graphical abstract

**Figure:**
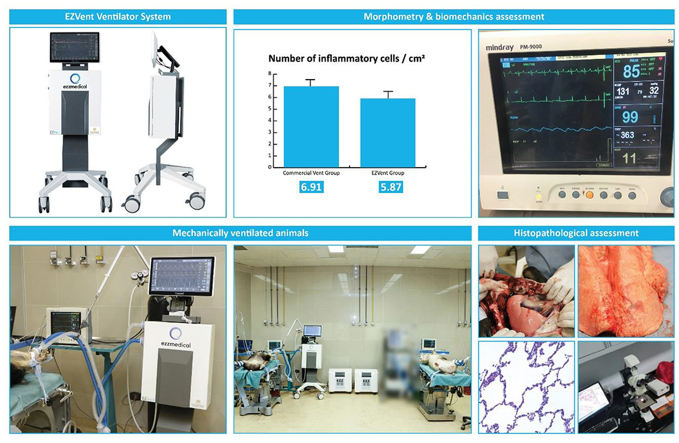

## 1 Introduction

Mechanical ventilation (MV) is the most popular short-term life support procedure globally [1]. A recent epidemiological study revealed that, around 310 persons/100,000 adult population are subjected to invasive ventilation for nonsurgical indications in USA [2]. Moreover, the global crisis caused by spread of COVID-19 during the last few years led to extraordinary and major demands on healthcare systems worldwide. Clinical presentation of COVID-19 ranges from flu-like symptoms to respiratory failure due to diffuse alveolar damage, the management of which required enhanced respiratory assistance and artificial ventilation. The increasing number of critically ill patients who are suffering from acute respiratory distress syndrome (ARDS)-induced respiratory failure, and the need for respiratory care and support, has been leading countries to rapidly establish new intensive care units (ICU) and try to provide affordable and practical solutions to manage the situation [3].

It is essential to ensure the safety and the performance of medical devices by applying all assessments required by the technical standards and, local and global applicable guidelines which state that the safety and efficacy of a device should be based on appropriate scientific research which can be inferred by well-controlled investigations [4, 5].

In the past, a major goal of MV was to ensure that patients had normal arterial blood gas levels, however, despite being a lifesaving procedure, researchers describe MV as: “a necessary evil”: which may coincide with significant latent complications [6]. Great efforts have been made over many decades to establish the concept of “protective ventilation” and currently, a priority is given to minimizing ventilation-induced lung injury (VILI), minimizing oxygen toxicity, and adjusting hemodynamics [7, 8]. García-Castro et al. [9], summarized the process of ventilators development in three phases: validation of the prototyping in high-trustworthiness clinical simulators, testing of it through experimental studies on animal models and lastly testing it on human. Therefore, the experimental studies on animals are important to provide initial evidence on the safety and performance of any developing ventilators and respiratory supportive devices. The lobar, and bronchial anatomy in addition to the lung histological structure of pigs have great similarities to that of human. So, the porcine model is widely considered the gold standard model for the evaluation of lung ventilators [9, 10, 11] and is recommended by some national health authorities [12].

Since ventilation-induced lung injury has become a major concern in the modern era of MV, the clinical goals of MV had greatly changed [13]. Clinicians prefer mechanical ventilators that offer advanced meticulousness to avoid VILI as well as being efficient and safe during practicing ventilation modes currently available on all invasive and noninvasive mechanical ventilators. The main modes of ventilation used worldwide, permit the clinician to set the fraction of inspired oxygen (FiO2), positive end-expiratory pressure (PEEP), and a specific variable (pressure or volume) [14, 15].

EZVent Ventilator System is designed for use on patients requiring respiratory support or mechanical ventilation. It is designed to deliver tidal volumes ranging from 250-2500 ml for adult, at pressure ranges from 10-60 cm H_2_O, hence allowing adequate treatment for patients with varying inspiratory reserve volume. Mechanical ventilators that offer advanced precision to avoid VILI are preferable, that is why the EZVent series operating parameters are adjusted incrementally by 1 unit, excluding inspiration: expiration ratio (I: E) which is adjusted by 0.5 units and for tidal volume which is adjusted by 50 ml. In the EZVent ventilation system, the lung peak pressure is protected via electrically controlled safety valve that can be adjusted from 16 to 80 cm H2O in addition to mechanical safety valve operating at 80 cm H_2_O approximately and PEEP level could be restored within few milliseconds if it slips below the set level in case of low lung compliance and resistance.

The objective of this study was to evaluate the safety and the performance of a newly developed mechanical ventilator; EZVent in comparison to a standard commercial ventilator in terms of hemodynamics, arterial blood gases (ABG) lung histopathology and inflammatory markers expression in a healthy porcine model using 3 different ventilation modes; Continuous Mandatory Ventilation – Volume Control Mode (CMV-VC), Continuous Mandatory Ventilation – Pressure Control Mode (CMV-PC) and Continuous Positive Airway Pressure – Pressure Support Mode (CPAP-PS).

## 2 Materials and methods

### 2.1 Animals

The study was performed on eight male healthy pigs (Genus Scrofa Domestica) (Pigs farm, Giza, Egypt), weighing 30–40 kg each and aged 20±2 months old. The animals were housed in an adequate shelter at 20–25 °C and 60–70 % humidity, acclimatized for 96 h before the experiment, and were fed a standard pig diet (a corn-soybean meal-based diet contains crude protein ratio of 14.5%) with free access to water. The animals did not undergo any previous research. They received continuous veterinary care during the whole study period and were cared for by three expert animal technicians. They were deprived of food for 12 h before anesthesia. The study protocol was approved by Institutional Animal Care and Use Committee (CU-IACUC) Cairo University, Cairo, Egypt (Protocol approval Number: CU III F 5321), September 2021. An Animal Welfare Assessment Grid (AWAG) was performed on each animal during the study period based on the following parameters: physical, environmental, psychological, and procedural events. Animals were euthanized under ketamine anesthesia, and all efforts were made to minimize suffering. At the end of the study, carcasses were subjected to incineration in the biological wastes’ incinerator of the Learning and Research Center (LRC), Cairo University.

### 2.2 Sample size calculation

The sample size calculation was performed using G∗Power software 3.1.9.2 [16] based on the primary variable. equation was as follows: effect size (Cohen's d) (based on previous published study) [10] = 3.094, α = 0.05, power (1-β) = 0.90

Total sample size = 8 and sample size per group = 4

The sample size was calculated using the following formula [17]:

$$n=2{\left[\frac{\left(Z_{\alpha /2}+Z_{\beta }\right)*\sigma }{\mu_{1}-\mu_{2}}\right]}^{2}$$

n = sample size, Zα/2 = 2.576 (The critical value that divides the central 99% of the Z distribution from the 1% in the tail). Zβ = 1.645 (The critical value that separates the lower 5% of the Z distribution from the upper 95%). σ = the estimate of the standard deviation of HOMA IR = 3.094. μ1 = mean before ventilation = 2.95. μ2 = mean after ventilation = 1.37 [10].

### 2.4 Ventilators

EZVent is a newly developed mechanical ventilator aiming at delivering therapeutic benefits in the initial care of patients requiring urgent ventilation either invasively or non-invasively. It is designed to operate for short-term stabilization for a few hours to months depending on the severity of the clinical condition and to be a stationary product suitable for service in hospitals and critical care situations to provide continuous positive pressure respiratory support to the patient using medical oxygen. It could compress medical air from either the internal compressor or through the gas delivery network within hospitals. This ventilation system can deliver tidal volume (Vt) ranging from 250-2500 ml, at pressure ranges from 10-60 cm H_2_O. Besides, it could demonstrate broad operating ranges to give healthcare professionals more freedom, when making clinical decisions. The device was calibrated in the Calibration Lab Systems & Biomedical Engineering Department, Faculty of Engineering, Cairo University, Egypt. Report number: K93-AM003-0321.

The commercial ventilation used in the current study is a full feature ventilator consisting of a breath delivery unit, a graphical user interface, and several optional accessories, including a compressor, a back-up power source (BPS), and three cart options. Depending on the patient ideal body weight (IBW), the appropriate patient circuit is attached to the ventilator system and the patient. When the system is operational and connected to the appropriate utilities, the ventilator system delivers sensitive, precise breaths to critically ill patients. This ventilator system is a dual-microprocessor-based, touch screen controlled, critical care ventilator intendeds to provide continuous ventilation for neonate to adult patients (with expanded NeoMode Option) or infant to adult patients (with no Option) who require either invasive ventilation or non-invasive ventilation. The commercial ventilator system includes software that is intended for patients with ideal body weight as low as 0.3 kg and provides the user with tidal volume from 25 ml. The operating range of both ventilation systems are shown in Table 1.

**Table 1:** A table showing the operating range of both the commercial and EZVent ventilators.

Item	Unit	Value			
		EZVent		Commercial vent	
**Lung peak pressure**	CmH_2_o	10–60		5–90	
**PEEP**	CmH_2_o	0–20		0–45	
**I:E ratio**	Ratio	3:1	1:8 Pediatric/1:10 Adult.	4:1	1:299 for adult and pediatric
**Respiratory rate**	CPM	6	70 all Modes 30 SIMV Mode	1	100 for all modes
**Tidal volume**	Ml	30	250 Pediatric	25	2500 For adult and pediatric
		250	2500 Adult		
**O2 concentration**	%	21–100		21–100	
**Trigger pressure**	CmH_2_o	1–20		0.1–20	
**Rise time**	%	0–20		1–100	
**Peak inspiratory flow**	L/min	2–100		3–150	
**Pressure support**	CmH_2_o	0–40		0–70	
**Modes**	Type	CMV-VC		n/a	
		CMV-PC		n/a	
		Assisted Volume Control		Assisted Mode	
		SIMV VC-PS		SIMV	
		CPAP-PS		SPONT	

PEEP: Positive end-expiratory pressure, I: E: Inspiration: Expiration ratio, CPM: Cycle per minute. A/C: Assist/Control mode, SPONT: spontaneous mode, SIMV: synchronous intermittent mandatory ventilation.

The commercial ventilator was used as a standard one for comparison. It is FDA approved, CE-marked, commercially, and widely used in USA and many European countries. It was chosen as its specs were almost the same as the specs of EZVent for appropriate comparison and obtaining unbiased results.

### 2.5 Experimental protocol and procedures

Pigs were anesthetized by intramuscular injection of ketamine (20 mg/kg for induction; 5–30 mg kg/h for maintenance), xylazine (1 mg/kg for induction; 1.5–6 mg kg/h for maintenance) and paralyzed with pancuronium (initial dose: 0.1 mg/kg followed by 0.1 mg/kg/hour). Intramuscular atropine (0.02 mg/kg) was used as preanesthetic agent to reduce salivation and bronchial secretions [18, 19] (all from Pharmaceuticals, EGYDRUG, Egypt). Animals were put in the supine position and oro-tracheal intubation with a 7.0–7.5 ID endotracheal tube was performed [10, 18], and randomly allocated into two groups in a blinded manner: commercial vent group: the animals of which were anesthetized and ventilated using a widely used commercial ventilator, and **EZVent group:** the animals of which were anesthetized and ventilated using EZVent ventilator. A balanced electrolytes solutions were infused as needed to prevent collapse and were pre-warmed to guard against hypothermia.

### 2.6 Baseline measurements

Before the start of mechanical ventilation (t = 0), all physiologic parameters were measured including:

• Vital Signs: Rectal temperature, pulse, arterial blood pressure, respiratory rate (RR)
• Arterial Blood Gases (ABG): partial arterial oxygen pressure (PaO_2_), partial arterial carbon dioxide pressure (PaCO_2)_, bicarbonate (HCO_3_) and arterial oxygen saturation (SaO_2)_.
• Electro Cardio Graph (ECG).

### 2.7 Ventilation procedure

**Figure 1:**
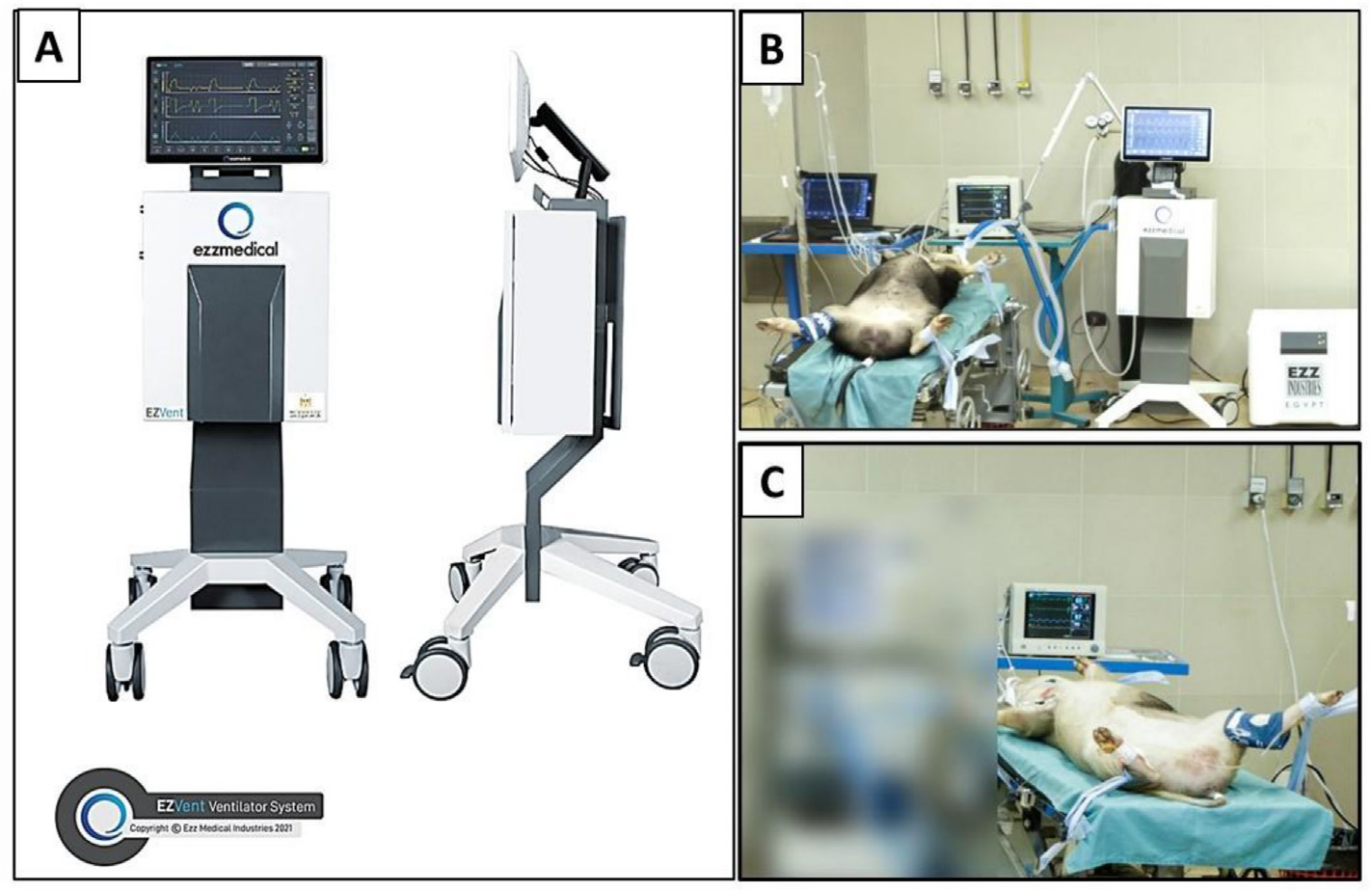
Photographs showing: A) EZVent ventilator. B and C) Two animals mechanically ventilated with EZVent and commercial ventilator, respectively.

The anesthetized animals were randomly assigned into two groups (n = 4 for each group). One group was ventilated by EZVent while, the other group was ventilated by the commercial ventilator (Figure 1). Three ventilation modes were tested each for 30 min on every animal at 3 different time intervals: **T30, T60**, and **T90**, respectively. The tested modes were: CMV-VC, CMV-PC and CPAP-PS, respectively.

The applied modes setting was as follows:

• CMV-VC: Tidal volume (Vt): 10 ml/kg, inspiration: expiration (I: E): 1:2, respiratory rate (RR): 15–20 BPM, fraction of inspired oxygen (FiO2): 40%, positive end expiratory pressure (PEEP): 4 cmH_2_O.
• CMV-PC: PIP: Pressure set to achieve Vt 10 ml/kg, I: E: 1:2, RR: 15–20 BPM, FiO_2_: 40%. PEEP: 4 cmH2O
• CPAP-PS: FiO_2_: 40%, CPAP: 4 cmH_2_O, pressure support: 10–15 cmH_2_O, trigger pressure: 2 cmH_2_O

### 2.8 Measurements taken in both groups

Vital signs (Temperature, pulse, arterial blood pressure, RR), lung mechanics (Plateau pressure, Vt, respiratory rate, and static compliance) and ABG (PaO_2_, PaCO_2_, HCO_3_ and O_2_ saturation) were taken at:

**T30**: after 30 min of CMV-Vc.

**T60**: after 60 min of CMV-PC.

**T90**: after 90 min of CPAP-PS.

### 2.9 Post-ventilation phase and histological assessment

After finishing the ventilation procedures, euthanasia of animals was carried out by intracardiac injection of thiopental (EIPICO, Egypt) overdose in a dose of 100 mg/kg. During necropsy and the removal of lungs, a continuous positive airway pressure equal to the PEEP was maintained to prevent alveolar collapse before or during fixation and to standardize the state of alveoli in all animals. The lung lobes were fixed by perfusion of 10% neutral buffered formalin at constant rate for appropriate fixation. Samples of lung tissue (5–7 cm^3^) were obtained from all the segments of each lobe according to its anatomical location [20], routinely processed, embedded in paraffin, sectioned into slices of 5 μm thickness, and stained with hematoxylin–eosin for histological analysis. For collecting representative samples, specimens from different segments of the cephalic, middle, and caudal lobes were obtained from both lungs; 10 non overlapping fields from cephalic and middle lobes and 25 fields from caudal lobe, based on their proportion of the total lung [21, 22], were examined by two independent pathologists who were blinded to the group identity. To identify VILI, a semi-quantitative scoring for alveolar emphysema, interstitial emphysema, atelectasis and inflammation response (scored from 0 to 4 each) was used according to the following scheme: 0 - no lesions, 1 - mild, focal lesions, 2 - mild, diffuse lesions, 3 - moderate lesions, and 4 - severe lesions [23, 24].

### 2.10 Western blot detection of TNF- α and IL-6 expression in lung tissue

The ReadyPrepTM protein extraction kit (total protein) supplied by Bio-Rad Inc (Catalog #163–2086) was utilized according to manufacturer instructions, Samples were harvested from the different lung lobes to be representative of the whole pulmonary tissue and homoginized. Lung samples from unventilated pig were used as a control. Protein concentration was measured using Bradford Protein Assay Kit (SK3041, Bio basic Inc, Markham Ontario L3R 8T4 Canada). Equal amounts of protein (20 μg) of each sample were loaded with an equal volume of 2x Laemmli sample buffer. Polyacrylamide gels electrophoresis were performed using TGX Stain-Free™ FastCast™ Acrylamide Kit (SDS-PAGE) from Bio-Rad Laboratories Inc Cat # 161–0181. Then, the blot was run for 7 min at 25 V to allow the protein bands transfer from the gel to the membrane using BioRad Trans-Blot Turbo. The membrane was blocked in tris-buffered saline with Tween 20 (TBST) buffer and 3% bovine serum albumin (BSA) at room temperature for an hour. The membranes were then incubated with an anti-TNF-α (ab96879) (1/1000 dilution) and IL-6 (ab6672) (1/500 dilution) antibodies. Primary antibodies were purchased from Abcam Inc (Waltham, MA, USA). Incubation was done overnight in each primary antibody solution, against the blotted target protein, at 4°C. Subsequently, the blots were washed in Tween 20-Tris buffered saline and then incubated in HRP-conjugated secondary antibody (Goat anti-rabbit IgG- HRP-1mg Goat mab -Novus Biologicals) solution against the blotted target protein for an hour at room temperature. The immunoreactive proteins were finally visualized using ECL Clarity TM Western ECL substrate Bio-Rad cat#170–5060) according to the manufacturer’s recommendation. The chemiluminescent signals were visualized and photographed using a CCD camera-based imager. Image analysis software was used to read the band intensity of the target proteins against control sample beta-actin (housekeeping protein) (Lot No. WL0001; Wanleibio, Beijing, China) by protein normalization on the ChemiDoc MP imager [25].

### 2.11 Immunohistochemical detection and localization of TNF-α and IL-6 in lung tissue

Paraffin-embedded tissue from different lung lobes of both lungs was cut into 5-μm sections. Immunohistochemical staining was made using the streptavidin-peroxidase method according to the manufacturer’s instructions for the used kits. Briefly, antigen retrieval was done by heating the slides in 10 mM sodium citrate in 0.05% Tween 20 at PH 6.0 followed by incubation of lung tissue in Universal Blocking Solution (Dako Corp., Carpinteria, CA, USA). Goat anti-mouse polyclonal antibodies against tumor necrosis factor- α (TNF-α) (Clone M-18, Santa Cruz Biotechnology, Santa Cruz, CA), and mouse monoclonal antibody against interleukin-6 (IL-6) (Abcam Inc,Waltham, MA, USA) were used as primary antibodies. Slides were incubated with primary antibodies followed by biotinylated secondary antibodies and visualized using diaminobenzidine (DAB). Counterstaining was performed with hematoxylin [26].

### 2.12 Morphometric assessment

Quantification of histopathological finding and morphometric analysis using Image J software “Image J 1.49 v/Java 1.6.0_244 (64-bit)" (National Institutes of Health, USA) were used to assess the following measures in both groups: area percentage of alveolar edema, alveolar wall thickening, and the mean number of inflammatory cellular infiltrate/cm^2^ of lung tissue [27]. Magnified H&E-stained slides (X400) were used for this purpose. Ten non-overlapping fields/each lobe of the cephalic and middle lobe and 25 fields/each caudal lobe were randomly selected and examined. The area percentage was assessed by using a calibration system that transforms the image pixels into micrometer. In immunohistochemically stained sections, field optical density was determined using the same software after subtracting the background noise. Six sections from each sample from all lobes were used to calculate the average positive TNF-α and IL-6 immunoactivity in lung tissue of both groups [28, 29].

### 2.13 Statistical analysis

Data were analyzed with Statistical Package for the Social Science (SPSS) 18.0 statistical software (SPSS Inc., Chicago, IL, USA). Numeric data with normal distribution were presented as mean ± standard error of mean (SEM). T-tests were used to compare continuous variables. P values < 0.05 were considered as a level of statistical significance.

## 3 Results

### 3.1 Mortality rate and adverse effects

All animals survived one and a half-hour of MV after which they were euthanized so no death cases were recorded during the procedures in both groups and no serious adverse effects (Pneumothorax, shock, hypotension, arrhythmia) were detected during the study for any animal.

### 3.2 Results of vital signs

Animal rectal temperature revealed no significant differences between EZVent group and commercial vent group throughout the study in all tested modes (p values ˃ 0.05). ECG monitoring showed normal sinus rhythm in both groups in all study time points including T30 (CMV-VC), T60 (CMV-PC), and T90 (CPAP-PS). Moreover, pulse and mean arterial pressure showed no statistically significant differences between the two groups at study time points T30 (CMV-VC), T60 (CMV-PC) and T90 (CPAP-PS) (p values ˃ 0.05). However, pulse showed statistically significant increase in EZVent group compared to commercial vent only at baseline (before the initiation of mechanical ventilation) (T = 0), (p = 0.028). The median values of both groups were in the acceptable normal ranges for the pulse in pigs with no major clinical significance **(**Table 2**)**.

**Table 2:** A table showing a comparative analysis of vital signs and ECG in all animals in both study groups during different moods using Wilcoxon rank-sum test.

Vital sign	Ventilation mode	Commercial vent group Mean ± SD	EZVent group Mean ± SD	p value
**Body temperature**	**Baseline (T = 0)**	36.75 ± 0.97	37.88 ± 1.54	0.2
	**CMV-Volume** **Control (T = 30)**	36.05 ± 0.72	37.27 ± 1.82	0.3429
	**CMV-Pressure Control (T = 60)**	35.60 ± 0.65	36.77 ± 2.04	0.3429
	**CPAP-PS (T = 90)**	35.20 ± 0.80	36.40 ± 2.12	0.5614
**Pulse (Beat/min)**	**Baseline (T = 0)**	80.75 ± 11.84	102.00 ± 19.04	0.028
	**CMV-Volume** **Control (T = 30)**	85.25 ± 17.25	98.00 ± 25.15	0.4857
	**CMV-Pressure Control (T = 60)**	84.75 ± 29.49	93.00 ± 27.89	0.4857
	**CPAP-PS (T = 90)**	75.25 ± 20.12	97.75 ± 17.90	0.2
**Blood pressure**	**Baseline (T = 0)**	88.33 ± 5.86	90.00 ± 12.53	0.849
	**CMV-Volume** **Control (T = 30)**	79.00 ± 18.19	70.00 ± 15.58	0.529
	**CMV-Pressure Control (T = 60)**	75.67 ± 23.46	71.50 ± 12.23	0.799
	**CPAP-PS (T = 90)**	113.00 ± NA	85.75 ± 19.81	0.306
**ECG**	**Baseline (T = 0)**	**Normal sinus rhythm NR** 4/4 (100.0%)	**NR** 4/4 (100.0%)	1
	**CMV-Volume** **Control (T = 30)**	**NR** 4/4 (100.0%)	**NR** 4/4 (100.0%)	1
	**CMV-Pressure Control (T = 60)**	**NR** 4/4 (100.0%)	**NR** 4/4 (100.0%)	1
	**CPAP-PS (T = 90)**	**NR** 4/4 (100.0%)	**NR** 4/4 (100.0%)	1

### 3.3 Arterial blood gases results

Assessment of PaO2, SaO 2, and HCO3 showed no statistically significant difference between EZVent group and commercial vent group at the evaluated time points: T0 (baseline), T30 (CMV-VC), T60 (CMV-PC), and T90 (CPAP-PS) (p values ˃ 0.05). No significant difference was detected between the PaCO2 values of EZVent group and commercial vent group in all modes. However, PaCO2 at T90 (CPAP-PS) showed a marginal difference (p = 0.042) **(**Table 3**).**

**Table 3:** A table showing a comparative analysis of arterial blood gases in all animals in both study groups during different modes using Wilcoxon rank-sum test.

Measured parameters	Ventilation mode	Commercial vent group Mean ± SD	EZVent group Mean ± SD	p value
**PaO2 (mmHg)**	**Baseline (T = 0)**	100.15 ± 19.18	90.83 ± 22.08	0.3429
	**CMV-Volume** **Control (T = 30)**	181.62 ± 31.53	159.47 ± 19.08	0.3429
	**CMV-Pressure** **Control (T = 60)**	178.35 ± 36.37	161.53 ± 43.93	0.8857
	**CPAP-PS (T = 90)**	177.55 ± 28.01	216.15 ± 77.26	0.8857
**PaCO2 (mmHg)**	**Baseline (T = 0)**	40.98 ± 7.09	34.30 ± 4.19	0.2
	**CMV-Volume** **Control (T = 30)**	26.75 ± 2.44	30.62 ± 3.64	0.2
	**CMV-Pressure** **Control (T = 60**	23.45 ± 2.34	27.73 ± 8.28	0.5614
	**CPAP-PS (T = 90)**	30.52 ± 4.67	41.48 ± 7.87	0.042
**HCO3 (mmol/L)**	**Baseline (T = 0)**	25.10 ± 3.78	22.93 ± 4.04	0.4857
	**CMV-Volume** **Control (T = 30)**	23.57 ± 2.22	23.88 ± 0.46	1
	**CMV-Pressure** **Control (T = 60**	23.23 ± 1.23	22.40 ± 2.90	0.6857
	**CPAP-PS (T = 90)**	25.98 ± 1.68	25.15 ± 1.68	0.3836
**O2 Saturation** **%**	**Baseline (T = 0)**	97.35 ± 1.03	96.88 ± 1.44	0.559
	**CMV-Volume** **Control (T = 30)**	99.53 ± 0.34	99.50 ± 0.58	0.8817
	**CMV-Pressure** **Control (T = 60**	99.53 ± 0.33	99.35 ± 0.94	0.8817
	**CPAP-PS (T = 90)**	99.50 ± 0.38	99.67 ± 0.47	0.655

### 3.4 Assessment of lung mechanics (plateau pressure, respiratory rate, static compliance and tidal volume)

Regarding Volume Control, Pressure Control and CPAP modes (T = 30, T = 60, and T = 90, respectively), no statistically significant differences were observed between EZVent and commercial control groups in all the measured lung mechanics parameters (Plateau pressure, static compliance, tidal volume, and respiratory rate) (p values ˃ 0.05) **(**Table 4**).**

**Table 4:** A table showing a comparative analysis of lung mechanics in all animals in both study groups during different modes using Wilcoxon rank-sum test.

Lung mechanics	Ventilation mode	Commercial vent group Mean ± SD	EZVent group Mean ± SD	p value
**Plateau pressure (cmH2O)**	**CMV-Volume** **Control (T = 30)**	17.50 ± 7.14	13.25 ± 2.99	0.4678
	**CMV-Pressure** **Control (T = 60)**	14.50 ± 1.29	15.75 ± 0.96	0.2338
**Respiratory rate**	**CMV-Volume** **Control (T = 30)**	21.75 ± 3.50	20.00 ± 4.08	0.6198
	**CMV-Pressure** **Control (T = 60**	38.25 ± 9.60	34.50 ± 3.51	0.3094
	**CPAP-PS (T = 90)**	29.75 ± 13.23	20.75 ± 4.86	0.3429
**Static compliance (ml/cmH2O)**	**CMV-Volume** **Control (T = 30)**	33.25 ± 8.62	39.00 ± 15.12	1
	**CMV-Pressure** **Control (T = 60**	23.23 ± 1.23	22.40 ± 2.90	0.6857
**Vt (ml)**	**CMV-Volume** **Control (T = 30)**	375.50 ± 57.02	355.00 ± 30.89	0.6857
	**CMV-Pressure** **Control (T = 60**	357.25 ± 114.26	363.00 ± 86.84	0.8857
	**CPAP-PS (T = 90)**	362.25 ± 87.57	480.50 ± 70.59	0.3429

### 3.5 Histopathological assessment of lung tissue

#### 3.5.1 Assessment of gross morphology

By inspection of all the animals, the following shared findings in all the study groups were recognized after opening the chest cavity: the two lungs and pleura were intact without any signs of rupture, shrinkage, or pneumothorax. The heart, pericardium and great vessels were seen in their normal anatomical sites occupying the middle and cephalic mediastinum without any detected abnormalities. The trachea in all animals bifurcated into two main bronchi and had an additional accessory bronchus leading to the right cephalic lobe arising from the trachea: about 0.5–1 cm proximal to its bifurcation.

**Figure 2:**
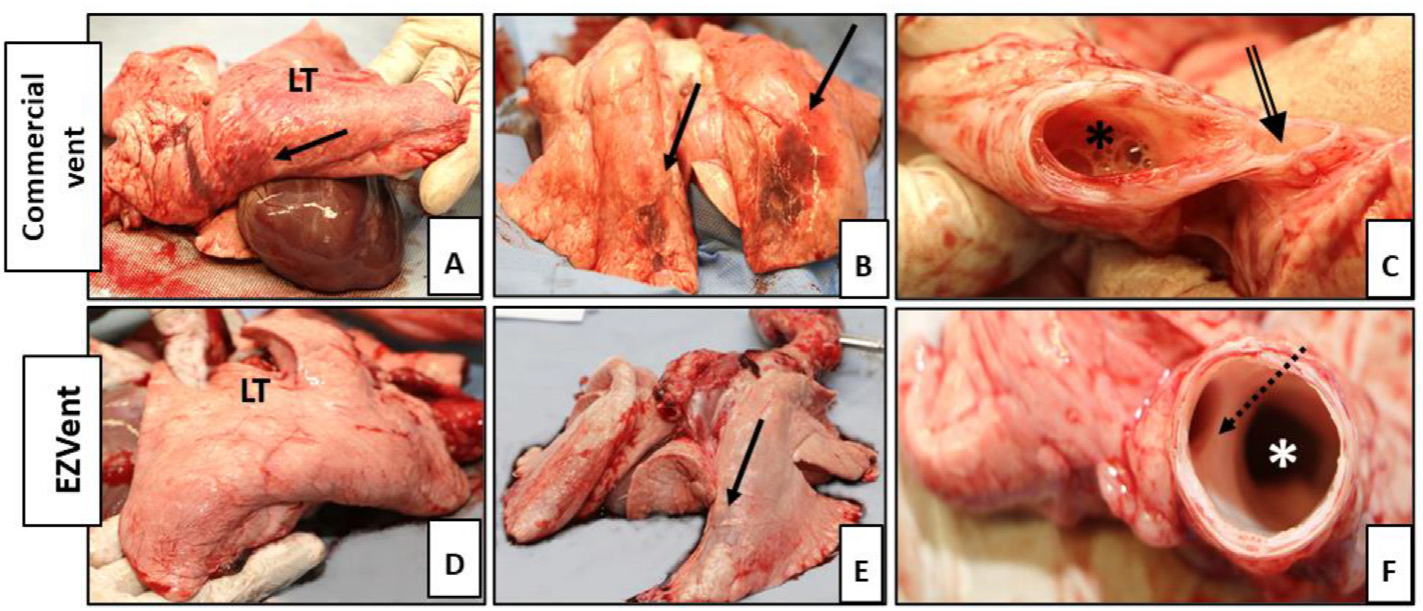
Photographs showing lung specimens' gross morphology of commercial vent group (A, B, C) and EZVent groups (D, E, F). The lung tissue showed congested lesions (arrow) of various severity: A & E showed minimal affection, B showed moderate affection, while D showed no affection. The lesions were noticed in the vertebral surface of caudal lobes. C: showed the site of carina masked with exudate (asterisk) and the entrance of the right cephalic bronchus (double arrow). F: showed the patent distal end of the trachea with clearly seen carina (dashed arrow) without any apparent exudate in the entrance of the main bronchi (asterisk). LT: left lung.

By examination of lung specimens of both ventilated groups, different gross morphology was detected, the lungs in the commercial vent group revealed some focal lesions of various size ranging from 1x1 to 2x3 cm mainly located in the caudal lobes of both lungs occupying its vertebral surface. The lesions appeared as congested patches or irregular areas of ecchymosis with a slightly firm consistency compared to the surrounding normal lung tissue. They were evident in almost all the 4 animals included in this group but with various severity (3 of them were moderately affected, and 1 was minimally affected). An animal of this group exhibited colorless watery exudate in the distal end of the trachea covering the carina. However, in the EZVent group, minimal affection was only recognized in the lung of two animals out of four. The detected lesions occupied only a small area of the vertebral aspect of the caudal lobes. Their color was lighter, and they were less firm in comparison to the lesions of the commercial vent group (Figure 2).

#### 3.5.2 Assessment of histological sections stained with H&E

**Figure 3:**
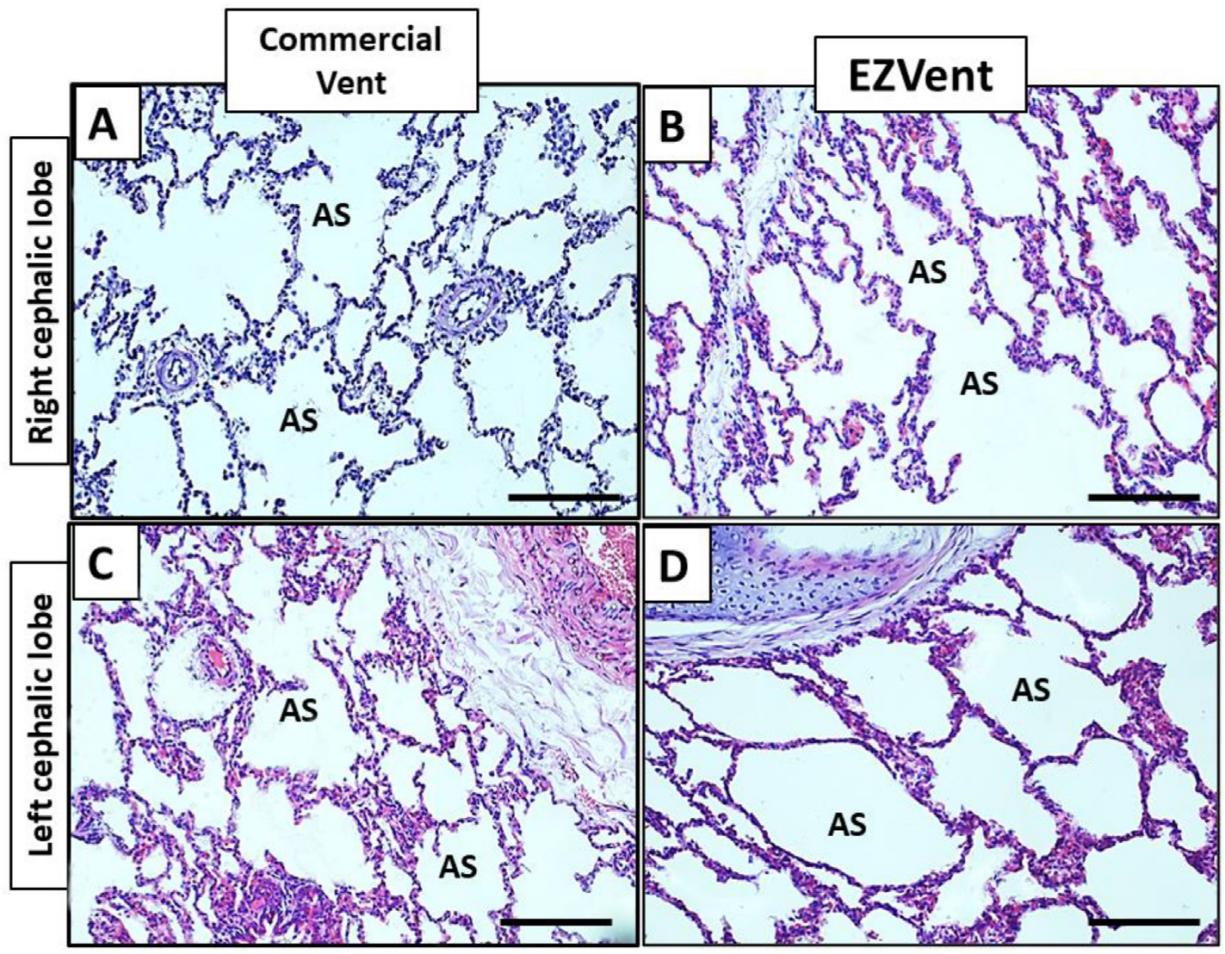
Photomicrographs showing histological sections of the right and left cephalic lobe of pig lung of commercial vent group (A, C) and EZVent group (B, D). Alveolar spaces (AS) were patent without any hemorrhage, inflammatory exudate, or oedema. The alveolar walls, interstitial tissue, bronchioles, and blood vessels showed normal histological structure. H&E scale bar: 50 μm in panel A and B, X20 and 20 μm in C and D, X40.

**Figure 4:**
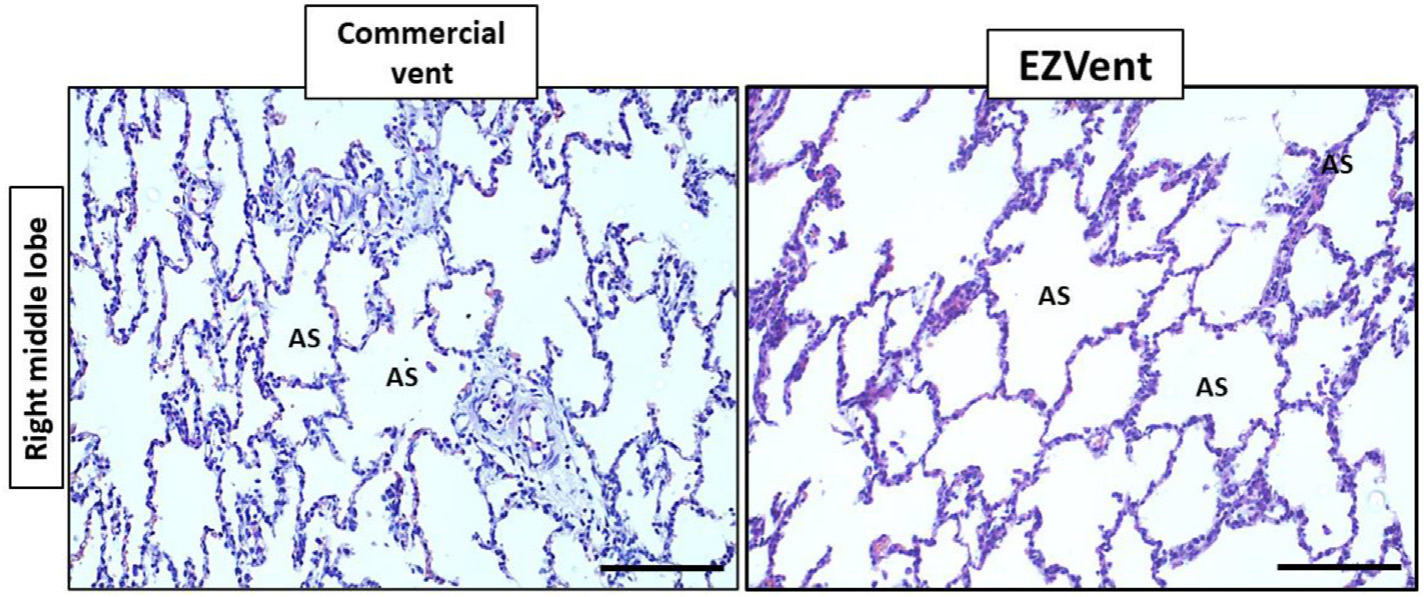
Photomicrographs showing histological sections of the middle right lobe of pig lung of commercial vent group and EZVent group. Alveolar spaces (AS) were patent without any atelectasis, hemorrhage, inflammatory exudate, or oedema. The alveolar walls, interstitial tissue, bronchioles, and blood vessels showed normal histological structure. H&E scale bar: 50 μm.

**Figure 5:**
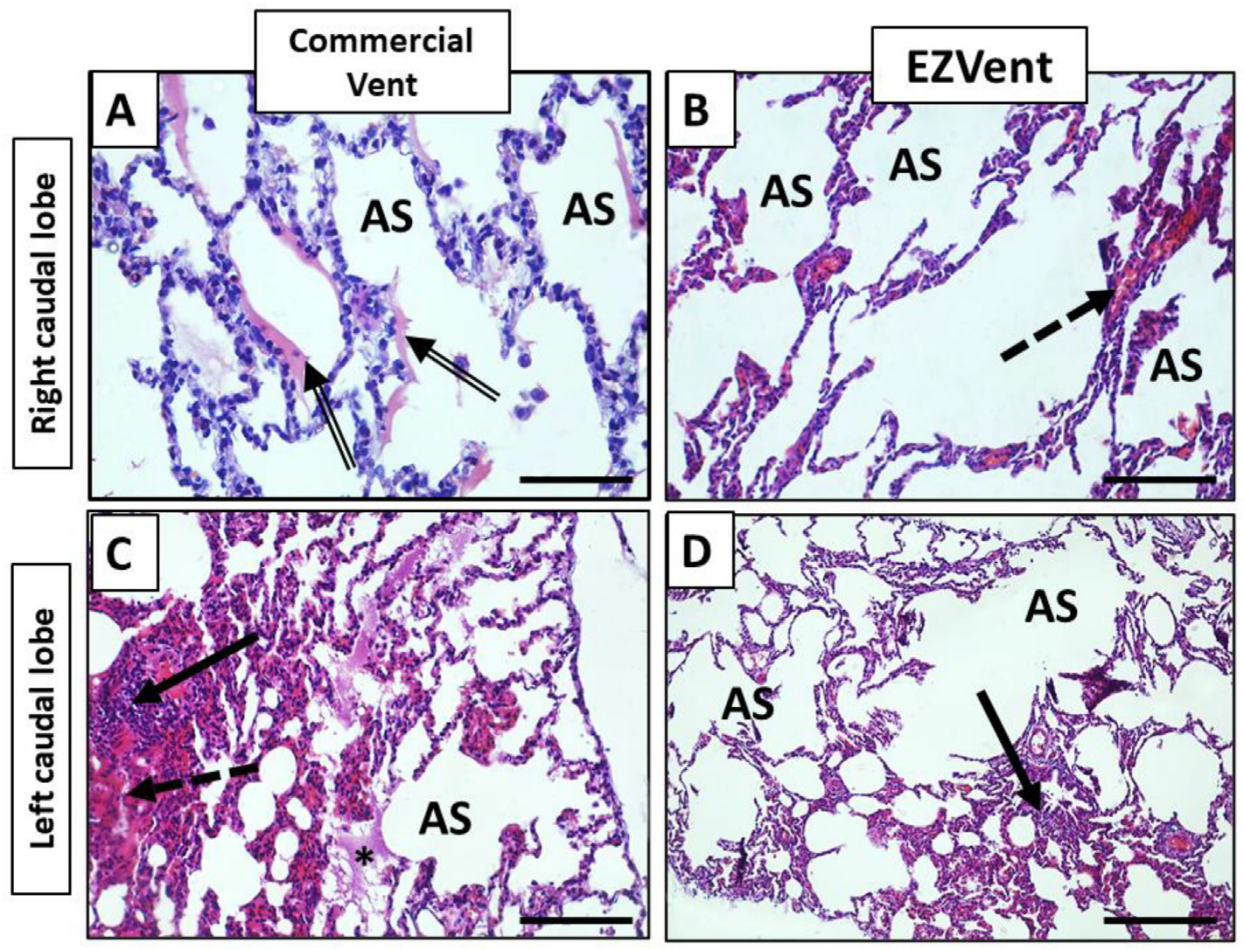
Photomicrographs showing histological section prepared from the macroscopically affected sections of the caudal right and left lobes of pigs in the commercial vent group (A, C) and EZVent group (B, D). Alveolar spaces (AS) showed no hemorrhage or inflammatory exudate. Focal lesions in a few segments of caudal lobes appeared in the form of hyaline membrane (double arrow), interstitial inflammatory cellular infiltrate (arrow), alveolar oedema (asterisk) and interstitial congestion (dashed arrow). H&E Scale bar: 20 μm in panels A and B, X40 and 50 μm in C&D, X20.

Histological section of lung tissue of commercial vent group:

There was no evidence of massive alveolar damage, degeneration of bronchial epithelium, atelectasis, fibrosis, chronic lesions, or consolidation in all lung lobes of this group. There was no remarkable microscopic histopathological alteration in the cephalic, middle, and accessory lobes of both right and left lungs (Figures 3 and 4). The caudal lobes of both lungs in almost all animals showed few focal histopathological changes in the form of few collapsed alveoli, alveolar wall thickness, septal oedema, alveolar oedema, interstitial neutrophil and microphages infiltrate, and minimal interstitial congestion. Hyaline membrane was noticed in few fields of the caudal lobe of left lung of only one animal (Figure 5). These findings have a focal pattern of distribution in the vertebral aspect of the caudal lobe and were only observed in the sections prepared from areas having changes in their macroscopic appearance. The remaining parts of this lobe and almost all segments of other lobes did not exhibit any histological abnormalities.

**Histological examination of the lungs in EZVent group** showed no detected abnormal histological structure of cephalic lobes of both right and left lungs as well as the middle and accessory lobe of right lung (Figures 3 and 4). The caudal lobe of both lungs in only two animals showed focal minimal interstitial congestion and neutrophil infiltrate with apparent alveolar wall thickening only in sections of areas that had altered macroscopic pictures (Figure 5). Collapsed alveoli were hardly seen in this group. Both lungs of all animals were obviously free of any alveolar rupture, atelectasis, fibrosis, focal lesions, chronic lesions, or consolidation.

#### 3.5.3 Histopathological scoring of lung tissue

Scoring of pulmonary tissue histopathology using a semiquantitative score (0–4) on blinded, randomly selected fields showed a non-significant difference between the study groups (p˂0.1). The evaluated items and the results of this score are shown in (Table 5); there was no significant difference between the control and the test group**.**

**Table 5:** A table showing pulmonary tissue histopathological score (mean ± SD) in all animals of the study groups.

Group	Alveolar emphysema	Interstitial emphysema	Atelectasis	Inflammatory response	p Value
**Commercial vent group**	0	0	0	0.52 ± 0.04	**0.1**
**EZVent group**	0	0	0	0.41±0.03	

#### 3.5.4 Morphometric analysis of histopathological finding

**Chart 1:**
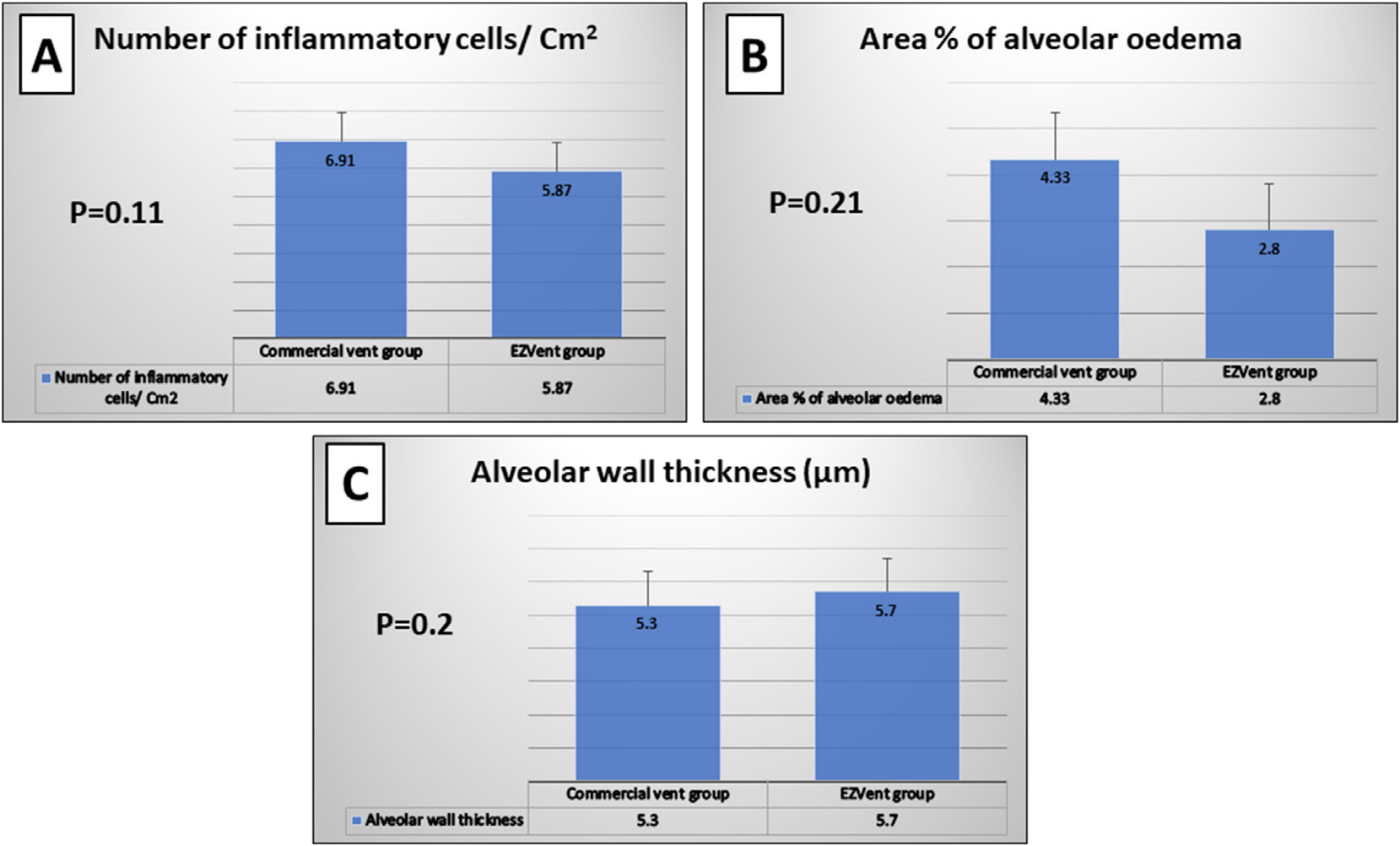
A bar chart showing mean ± SD of the number of inflammatory cell infiltrate, area percentage of alveolar oedema, and alveolar wall thickness (μm) in all animals of all groups.

Number of inflammatory cells/cm^2^ in high power field (X40):

Although there was an apparent increase of cellular infiltrate in few sections of caudal lobes of both ventilated groups, morphometric quantification of number of cells/cm^2^ and statistical analysis of this data revealed a non-significant difference in their number between the two groups (p = 0.11) **(**Chart 1 A**)**.

Area percentage of alveolar oedema:

The mean area percentage of alveolar oedema was proved to be statistically non-significant (p = 0.21) when comparing the control and the test groups (Chart 1 B).

Alveolar wall thickening:

The alveolar wall thickness of the lung in both ventilated groups apparently increased in the histological sections of a few segments of the caudal lobe, but this increase had no statistical significance (p = 0.2) (Chart 1 C).

#### 3.5.5 Assessment of TNF-α and IL-6 expression in lung tissue

**Figure 6:**
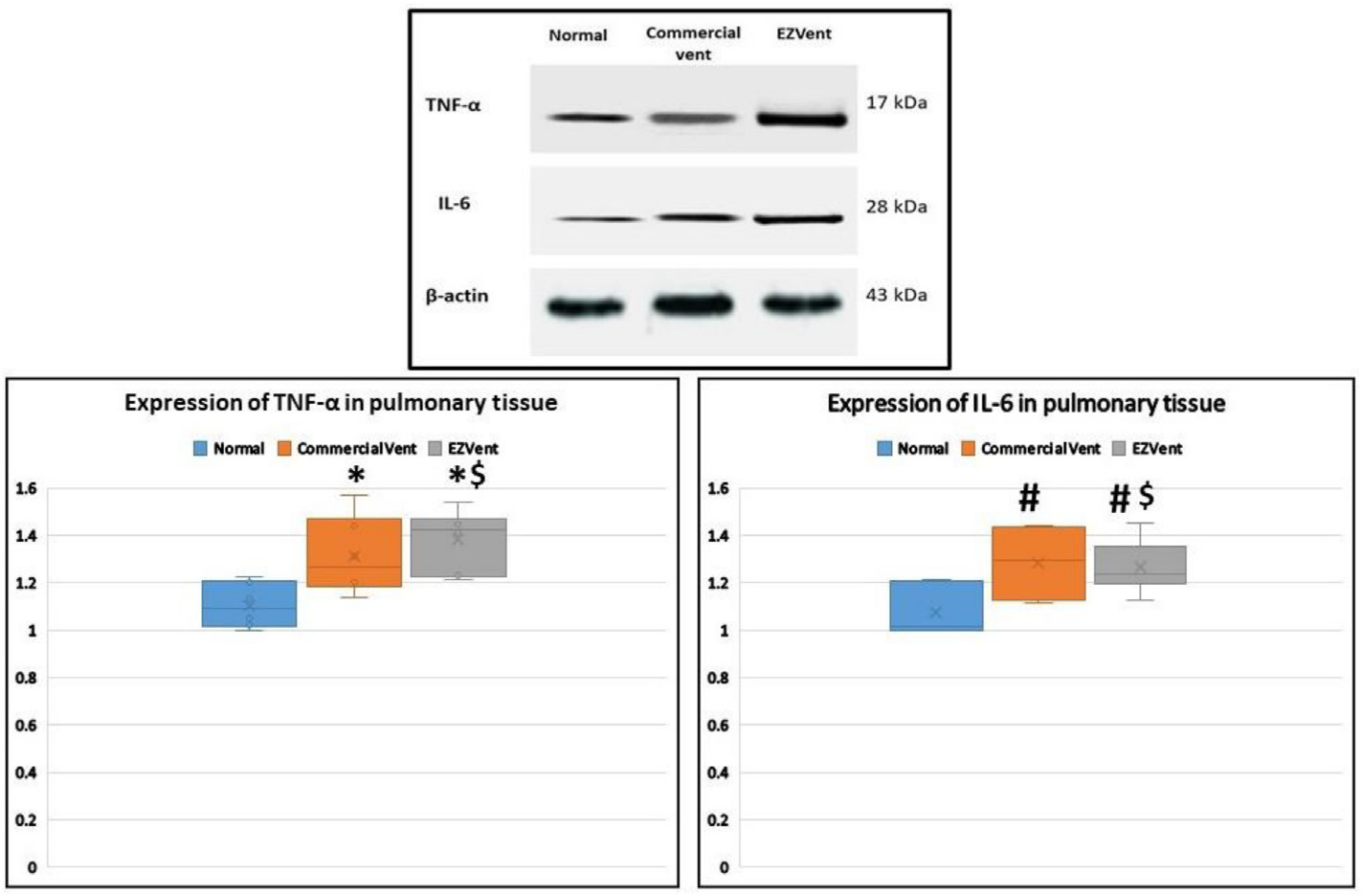
Representative image and statistical box blots of Western blotting of pulmonary tissues for detecting the protein expressions of TNF-α and IL-6. Normal group indicates lungs of unventilated pigs. N = 6. ∗: p = 0.3 compared to normal group; #: p = 0.1 compared to normal group; and $: p = 0.5 compared to commercial vent group. The original figures of gel and blot are available as Supplementary Figure S1.

Western blot analysis of TNF-α and IL-6 proteins expression in pulmonary tissue of the study groups revealed a non-significant increase of their expression in the lung tissue of both ventilated groups compared to the normal non-ventilated lungs ((p values ˃ 0.05) (Figure 6). The original blots were provided in supplementary data as Supplementary Figure S1.

#### 3.5.6 Localization of TNF-α and IL-6 in lung immunohistochemically stained sections

**Figure 7:**
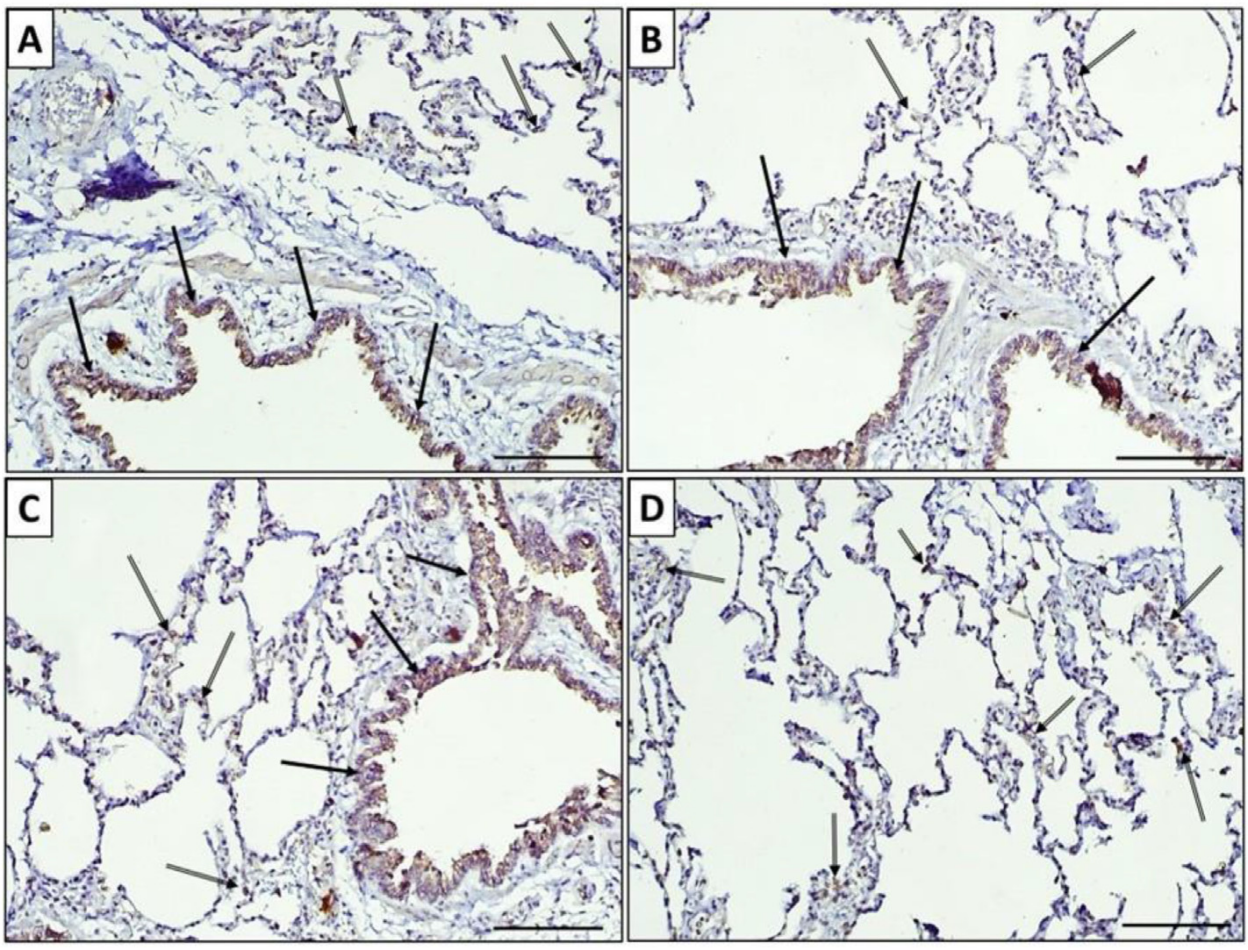
Photomicrographs showing histological sections of pig lung, immunohistochemically stained with TNF-α Antibody. Lung sections of commercial vent group (A and B) and EZVent group (C and D) showed moderate immunoactivity in the epithelial cells lining the bronchial tree (arrow) and in a few cells of the alveolar septa (double arrow). The positive reaction appeared as cytoplasmic brownish discoloration. Scale bar: 50 μm.

**Figure 8:**
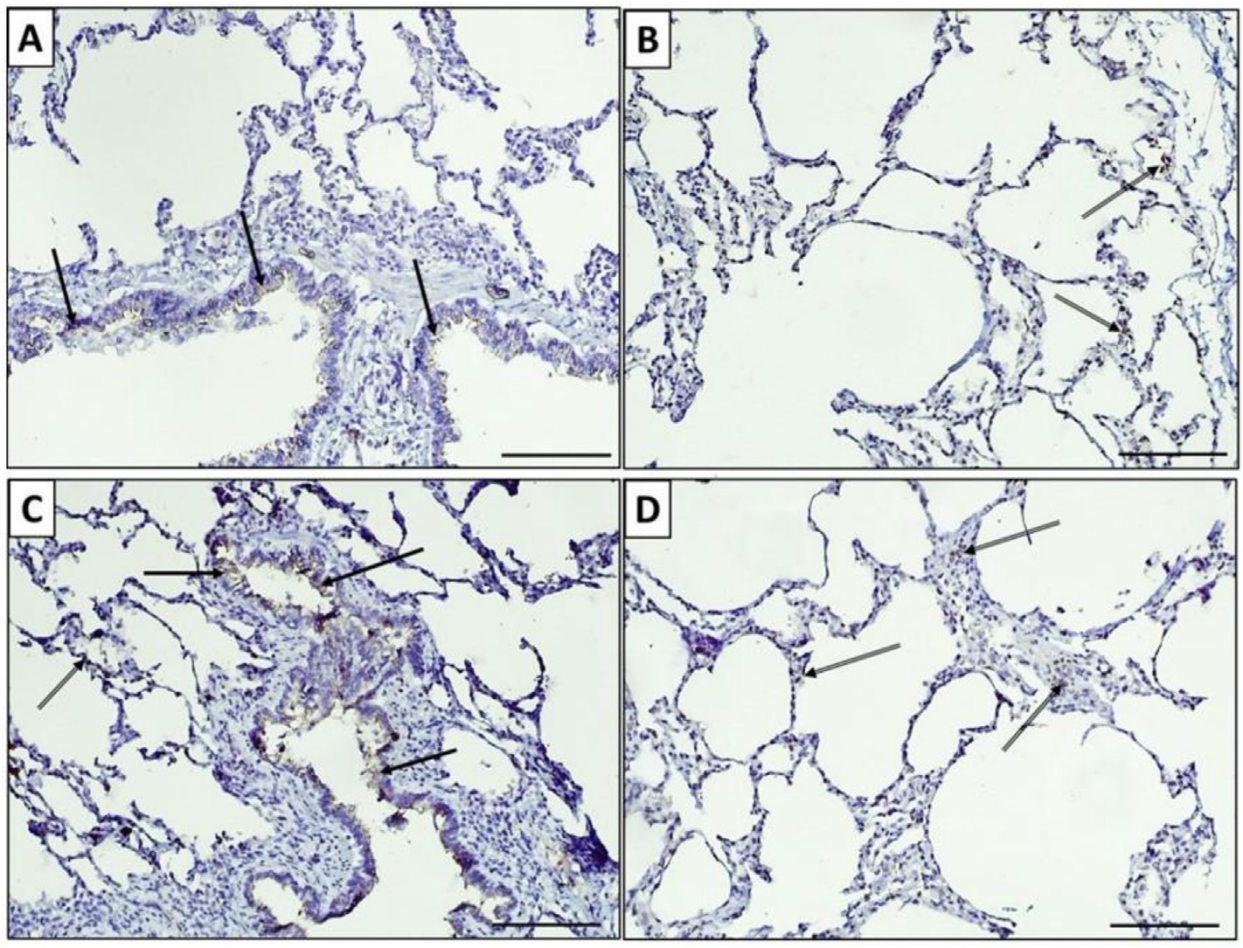
Photomicrographs showing histological sections of pig lung, immunohistochemically stained with IL-6 Antibody. Lung sections of commercial vent group (A and B) and EZVent group (C and D) showed minimal immunoactivity in the epithelial cells lining the bronchial tree (arrow) and in a few cells of the alveolar septa (double arrow). The positive reaction appeared as cytoplasmic brownish discoloration. Scale bar: 50 μm.

**Figure 9:**
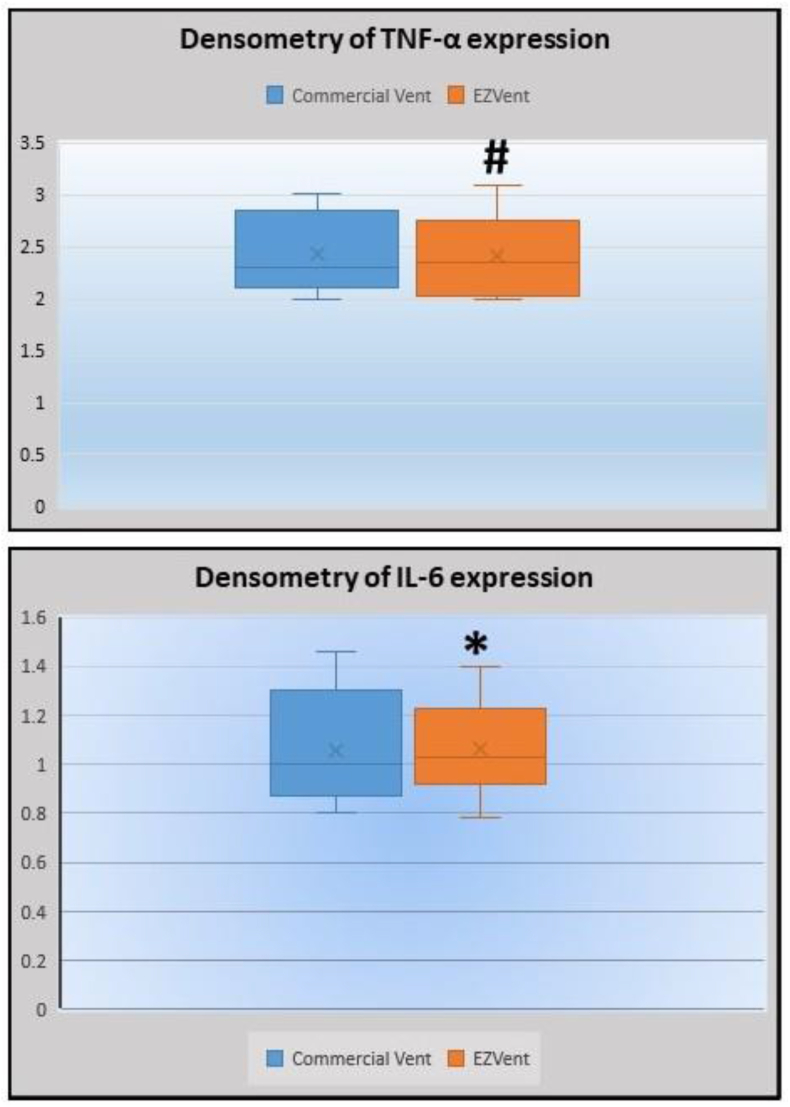
Box plots showing the mean optical density of TNF-α and IL-6 immunoactivity in pig lungs of the study groups. ∗: p = 0.1 compared to commercial Vent group; #: p = 0.2 compared to commercial Vent group.

The lung TNF-α and IL-6 immunohistochemically stained sections of both ventilated groups showed minor cytoplasmic immunopositivity in the epithelial layer lining the bronchioles in addition to few cells within the alveolar septa and interstitial spaces. The positive expression of these markers was obviously more abundant in the lining epithelium of the bronchial tree than in the alveoli themselves (Figures 7 and 8). Morphometric quantification of this data displayed a non-significant difference between the commercial vent and EZVent group regarding the optical density of both TNF-α and IL-6 immunoactivity in lung sections (Figure 9).

## 4 Discussion

A newly developed ventilator system (EZVent) was compared to a traditional standard commercial widely used ventilator regarding their efficiency to establish putative levels of arterial blood gases and lung mechanics along with the assessment of their effect on lung histological structure in a healthy porcine model. The specifications and operating range of the tested ventilators systems were almost comparable. Both were designed for respiratory support in hospitalized mechanically ventilated patients to provide them with continuous positive pressure respiratory support.

The porcine model was selected in the current study as it was globally approved to be the ideal model for evaluation of lung ventilators [9, 10, 11], broadly used and recommended in translational respiratory research [6] due to the large resemblances between its lung and the human lung [20]. In several studies, the pig model was commonly used to assess the newly developed ventilators and respiratory support devices to ensure their safety and performance compared to a commercial-approved devices [9, 11, 30, 31, 32].

Our results revealed no statistically significant difference between EZVent group and commercial vent group regarding PaO2, SaO 2, and HCO3 at the evaluated time points: T0 (baseline), T30 (CMV-VC), T60 (CMV-PC), and T90 (CPAP-PS). PaCO2 values differences also were insignificant in all modes between EZVent group and commercial vent groups. However, PaCO_2_ at T90 (CPAP-PS) showed a marginal difference (p = 0.042). This difference was clinically not significant as the reported PaCo2 values for both groups were consistent with the pigs’ known reference range values [33, 34]. This difference can be explained by the effects of the animal conditions during the last phase of the study (CPAP), in which the animal starts to breathe spontaneously after relief from the dose of anesthetics. The difference in the response to anesthesia and the time to achieve full awakening between animals may result in variation of respiratory drive and hence CO2 elimination, explaining the difference between the two groups.

During volume control, pressure control, and CPAP modes (T = 30, T = 60, and T = 90, respectively), there were no statistically significant differences observed between EZVent and commercial control groups in all the measured lung mechanics parameters (Plateau pressure, static compliance, Vt, and respiratory rate). Furthermore, assessment of vital signs revealed that animal rectal temperature showed no significant differences between EZVent group and commercial vent group throughout the study in all tested modes. ECG monitoring showed normal sinus rhythm in both groups in all study time points T30 (CMV-VC), T60 (CMV-PC), and T90 (CPAP-PS). In addition, pulse and mean arterial pressure showed no statistically significant differences at the same time points. Pulse showed a statistically significant increase in EZVent group compared to commercial vent only at baseline (Before the initiation of mechanical ventilation) (T = 0), The median values of both groups were within the acceptable normal ranges for the pulse in pigs with no major clinical significance.

Our results revealed a non-significant difference between the commercial vent group and EZVent group regarding pulmonary histopathological semi-quantitative score, area percentage of alveolar oedema, number of inflammatory cells/cm^2^, and mean of alveolar wall thickness. Histopathological findings are consistent with the reported effects of mechanical ventilation in animal models [8, 24, 30].

In the current research, a minimal degree of the altered histological structure was recognized in few lungs of both study groups with no statistical difference between them and this was not reflected in the overall lung histopathological score or the clinical picture of the animals as it was very localized affecting very few segments of the dependent areas of the lungs (vertebral aspect of the caudal lobes). The affected lung tissue appeared as congested lesions if measured, won’t exceed 0.5–1% of the total lung tissue by using different methods of lung lesion scoring systems [22, 23].

Our results revealed a non-significant difference between the commercial vent group and EZVent group regarding pulmonary histopathological semi-quantitative score, area percentage of alveolar oedema, number of inflammatory cells/cm^2^, and mean of alveolar wall thickness. Histopathological findings are consistent with the reported effects of mechanical ventilation in animal models [8, 24, 30]. In a previous study on healthy mice ventilated using different peak pressure (low peak pressure: = 20 cm H_2_O and high peak pressure: = 40 cm H_2_O), even the mice of the low peak pressure group were subjected to ventilation using clinically relevant ventilation setting, lung histopathology examination revealed, edema, inflammatory cell infiltration, alveolar hemorrhage, and alveolar wall thickness in lungs from both high–peak pressure and low peak pressure groups compared to the control nonventilated animals [35]. In the same study, microvascular permeability index was analyzed after performing Evans blue dye and showed a significantly higher vascular permeability in both ventilated groups as compared with normal nonventilated mice. This may explain the histopathological findings of alveolar and septal oedema.

The estimated pulmonary histopathological score in the current study in both ventilated groups was 0.52 in commercial vent group and 0.41 in EZVent group. It showed lower values than those recorded by Wolthuis et al. [33], who reported that MV attributed to VILI in mice, without a priming pulmonary affection, even with the usage of relevant ventilator settings. They compare using two different Vt; low and high Vt (7.5 ml/kg and 15 ml/kg, respectively) for 5 h and recorded a VILI score of 1.0 and 2.0 in Low and high Vt groups, respectively. Moreover, in the above-mentioned study of Belperio et al. [35], MV for 6 h resulted in a pulmonary histopathological score of 2.5 and 7.5 in low–peak pressure and high-peak pressure groups, respectively [36]. These values were higher than the values recorded in the current study even with using almost all the same parameters of the histopathological score including alveolar congestion, hemorrhage, leukocyte infiltration, or aggregation of neutrophils in airspace or the vessel wall, and thickness of the alveolar wall. This may be due to species differences, or the longer ventilation period used in the aforementioned studies.

The histopathological lesions noticed in the current research had two main microscopic features; the first was the macrophages and neutrophilic infiltrate which resulted in alveolar wall thickness. Simply, these inflammatory changes observed in healthy lungs could be explained by physiological adaptations to the artificial process of MV and despite its presence, it was coincident with the preservation of the pulmonary tissue architecture. This finding is in accordance with previous research on mice, where low clinically relevant Vt was used for short term ventilation and was proved to cause a reversible inflammatory reaction, while maintaining tissue integrity [37, 38]. In the study of Pastore et al. [23] two groups of healthy pigs were ventilated using Vt of 8 and 20 ml/kg for 240 min and were compared to a 3^rd^ group of spontaneously breathing pigs. They reported that MV strategies affect lung parenchyma integrity and functionality and induce a local inflammatory response in healthy pig lungs detected by increased lung content of MMP-9 (Matrix metalloproteinase-9) of both mechanically ventilated groups compared to the spontaneous breathing animals, because of neutrophils and macrophages recruitment in lung tissue. Moreover, they reported that the complete activation of MMP-2 in other organs including kidney and liver, as a systemic inflammatory reaction was obviously seen in the 20 ml/kg Vt group and minimally in the 8 ml/kg Vt group after a 240 min ventilation period.

The second histopathological finding noticed in the current research was alveolar oedema and interstitial congestion which was recognized only in the dependent areas of lung tissue. This could be explained by the positive pressure mechanical ventilation that may direct unequal distribution of air between the lung areas being more in the upper and ventral independent region especially with the absence of diaphragmatic contraction. Such mechanical stress and strain were found to impair the endothelial, interstitial, and epithelial integrity of healthy lungs, moreover, the endothelium was proved to be impaired within 5 min of MV [37, 38, 39]. In a previous study, similar congested lesions in the dependent areas of healthy sheep lungs were detected by means of computerized tomography. These lesions appeared as dense areas and their microscopic examination revealed congestion and atelectasis [40].

On the other hand, the lung independent areas in the current study showed no evidence of any signs of VILI, indicating appropriate ventilation, aeration, and pressure as it was described in many studies that these areas are more vulnerable to barotrauma than the lung-dependent areas and the affection may range from rupture of few alveoli to formation of lung cysts, multiple cysts, bronchiectatic changes, and even pneumothorax or pneumomediastinum [38, 41]. Fortunately, all these barotrauma consequences were excluded in the current research by both inspection of lungs in situ and their histopathological examination.

It is worth mentioning that the control nonventilated, spontaneously breathing, anesthetized animals in some experiments of VILI in healthy animals exhibited histopathological alteration of their lungs in the form of acute alveolar emphysema, interstitial emphysema, atelectasis, and inflammation (score 1) along with increased pulmonary arterial pressure [23]. This may be triggered by, at least in part, difficult breath caused by the non-physiological supine position in which the animals were positioned during the experimental procedure, and the inhibitory effect on respiratory function and loss of muscle tone, as caused by muscle relaxants, anesthetics, and sedatives such as thiopental sodium. It was also reported that high oxygen concentration in inspired gas during MV is a prerequisite to produce atelectasis in the healthy lungs during anesthesia [42].

In the current study there was a non-significant increase of proinflammatory cytokine TNF-α and IL-6 expression in lung homogenates of both ventilated groups compared to the control values measured in a normal non ventilated lung and without a significant difference between them. However, In the study of Pastore et al. [23], it was proved that pulmonary levels of tumor necrosis factor (TNF)- α, interleukin (IL)-6, keratinocyte-derived cytokine (KC), and macrophage inflammatory protein (MIP)-2 in lung tissue homogenate of ventilated healthy pigs increased significantly compared to the control lungs. This significant difference between the ventilated and control lungs may be caused by the longer ventilation duration (240 min). On the other hand, Altemeier et al [43] verified that MV using clinically relevant tidal volume for 6 h did not cause significant cytokine expression. In Altemeier et al’ study, the cytokines were measured in the bronchoalveolar fluid not the pulmonary tissue itself and this was assumed to be attributed to localization of cytokines in the sub-epithelium and incapability to migrate further into the alveoli, hence, could not be secreted in the bronchoalveolar fluid. Our immunohistochemical results regarding the localization of TNF-α and IL-6 in pulmonary tissue may support this assumption as the positive immunoactivity was expressed more in the bronchial epithelium and to a lesser extent in the alveolar walls. In other words, we could suppose that the lining epithelium of the bronchial tree may be the first population of pulmonary cells that tend to be affected by VILI.

The limitations of the current study include a relatively short period of ventilation and a lack of facilities and equipment in the veterinary surgical room necessary for invasively assessing the pulmonary blood pressure, the wet/dry weight ratio of the lungs to detect pulmonary oedema, and esophageal pressure as an indicator for pleural pressure during ventilation. However, this study provided an initial screening for a newly developed ventilator considered for mass use and proved that EZVent can offer sufficient gas exchange for one and a half-hour with minimization of VILI, minimal inflammatory response, and stable hemodynamics, however, we recommend further study on human prior to large-scale procurement of this ventilator.

## 5 Conclusion

This study provided trusted evidence regarding the equivalence of EZVent to other commercial ventilators. The difference in hemodynamics, lung mechanics, gas exchange, pulmonary histopathological scoring, morphometric analysis, and cytokines expression in pulmonary tissue of the mechanically ventilated animals of both groups did not differ significantly which confirms the safety and the efficacy of EZVent. Still, the measured parameters need to be re-tested for a longer ventilation period. In addition, testing the ventilator on humans prior to mass use is recommended.

## Declarations

### Author contribution statement

Kamal Hussei, Ahmed F. Ahmed, Magda M.A. Omar and Rania A. Galhom: Conceived and designed the experiments; Performed the experiments; Analyzed and interpreted the data; Wrote the paper.

Mostafa Salah and Ola Elrouby: Conceived and designed the experiments; Analyzed and interpreted the data; Contributed reagents, materials, analysis tools or data.

Yasser Nassar: Conceived and designed the experiments; Analyzed and interpreted the data.

### Funding statement

This work was supported by EZZ Medical Industries, Egypt.

### Data availability statement

Data will be made available on request.

### Declaration of interest’s statement

The authors declare no conflict of interest.

### Additional information

No additional information is available for this paper.

## Appendix A Supplementary data

new collected blots and gel.jpg new collected blots and gel.jpg
