# Supplementary figures and images for: Assessment of hemodynamics, blood gases, and lung histopathology of healthy Pig model on two different mechanical ventilators
Source: Heliyon. 2022 Sep 22;8(9):e10736. doi: 10.1016/j.heliyon.2022.e10736 (PMC9493143; doi:10.1016/j.heliyon.2022.e10736)

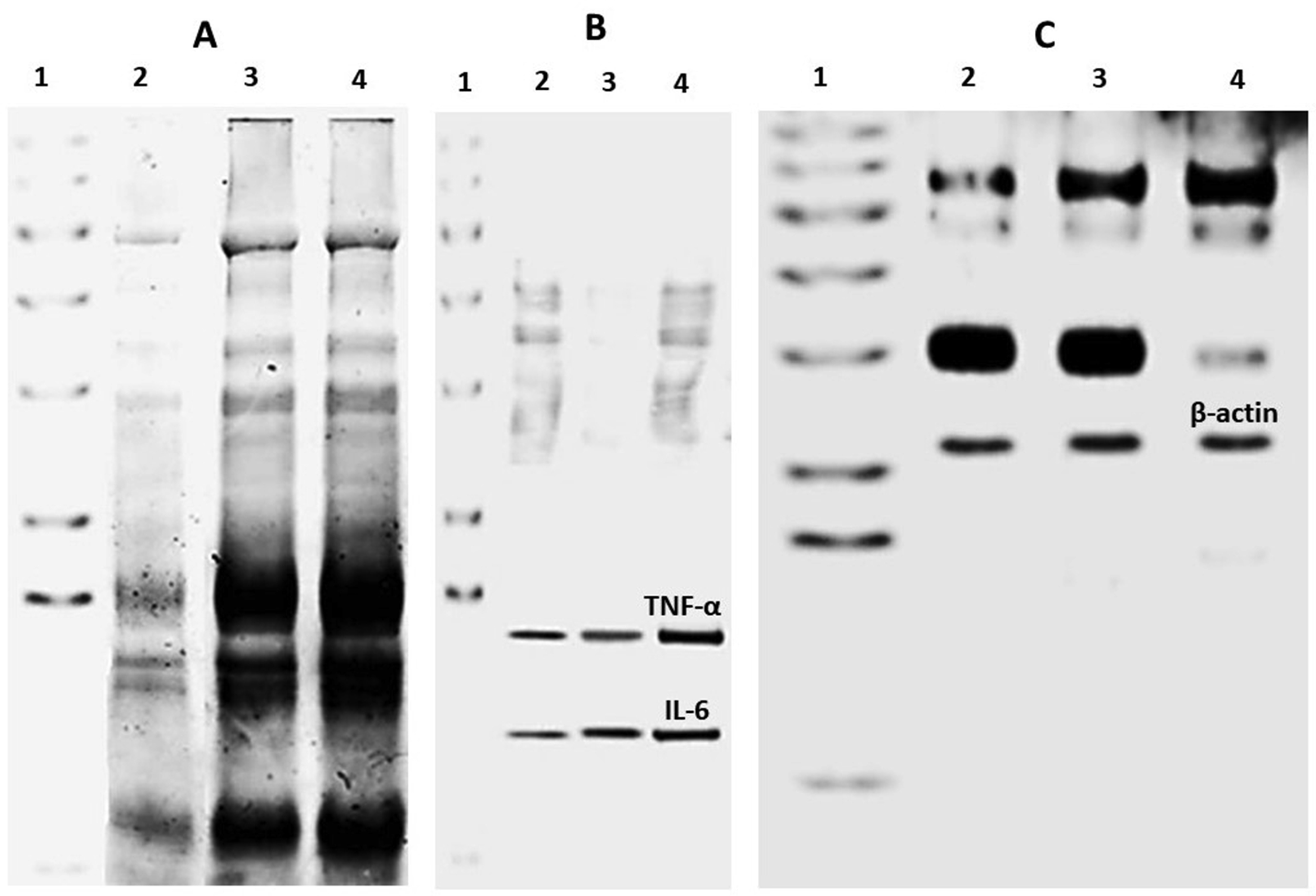

Supplement: new collected blots and gel.jpg [file figs1.jpg]
